# Mini-UAV Based Sensory System for Measuring Environmental Variables in Greenhouses

**DOI:** 10.3390/s150203334

**Published:** 2015-02-02

**Authors:** Juan Jesús Roldán, Guillaume Joossen, David Sanz, Jaime del Cerro, Antonio Barrientos

**Affiliations:** 1 Centre for Automation and Robotics (UPM-CSIC), José Gutiérrez Abascal 2, 28006 Madrid, Spain; E-Mails: jj.roldan@upm.es (J.J.R.); d.sanz@upm.es (D.S.); j.cerro@upm.es (J.C.); 2 ENSTA ParisTech, Boulevard des Maréchaux, 828, 91120 Palaiseau, France; E-Mail: guillaume.joossen@ensta-paristech.fr

**Keywords:** greenhouse, UAVs, sensory system, environmental monitoring, agriculture, robotics

## Abstract

This paper describes the design, construction and validation of a mobile sensory platform for greenhouse monitoring. The complete system consists of a sensory system on board a small quadrotor (*i.e.*, a four rotor mini-UAV). The goals of this system include taking measures of temperature, humidity, luminosity and CO_2_ concentration and plotting maps of these variables. These features could potentially allow for climate control, crop monitoring or failure detection (e.g., a break in a plastic cover). The sensors have been selected by considering the climate and plant growth models and the requirements for their integration onboard the quadrotor. The sensors layout and placement have been determined through a study of quadrotor aerodynamics and the influence of the airflows from its rotors. All components of the system have been developed, integrated and tested through a set of field experiments in a real greenhouse. The primary contributions of this paper are the validation of the quadrotor as a platform for measuring environmental variables and the determination of the optimal location of sensors on a quadrotor.

## 1. Introduction

Greenhouse farming is one of the most suitable areas for employing automation, robotic and computing technologies. Many greenhouses have climate control systems, which are usually composed of temperature and humidity sensors as well as irrigation, ventilation, and heating systems. These technologies offer a wide range of possibilities including climate control, production monitoring or detection of infestations or weeds. However, they suffer from several limitations, primarily due to their cost and reliability issues, which can make their implementation unprofitable and complex.

The emergence of Wireless Sensor Networks (WSNs) has initiated a revolution in these types of projects: WSNs provide flexibility (*i.e.*, the network can be constructed without a fixed architecture), modularity (*i.e.*, the network can incorporate new devices) and fault tolerance (*i.e.*, the network can work with failures in some motes) with low power consumption (*i.e.*, the motes usually have a sleep mode) to facilities [1].

Thus, these networks have been used in many applications in fields related to agriculture and food [2,3]: environmental monitoring [4] (e.g., climate monitoring and fire detection), precision agriculture [5] (e.g., rationalization of chemical products and optimization of irrigation) or the food industry (e.g., quality control and product traceability).

However, in the context of greenhouse farming, WSNs have been implemented more experimentally than productively. The previous literature contains several proposals concerning WSN deployment in greenhouses, but they are restricted to small fields [6]. For example, [7–9] deployed WSNs with nodes that measured temperature, humidity or luminosity in small greenhouses.

Some of the limitations of WSNs in greenhouse farming are the fixed locations of the motes and the corresponding costs, particularly for large greenhouses. Two possible alternatives to WSNs, which solve the problems of movement and costs, are mobile ground or aerial robots, which have been partially tested in previous studies [10–12].

Unmanned Aerial Vehicles (UAVs) are used in diverse fields related to environmental monitoring, such as in the acquisition of meteorological information [13,14]; the measurement of greenhouse gases in the atmosphere, which primarily includes carbon dioxide, methane and water vapor [15]; the surveillance of clouds of contaminant gases produced by human activities [16]; and the mapping of distribution of different gases [17].

In the context of agriculture, UAVs are typically used in some precision agriculture (PA) tasks: the measurement of vegetation density [18], the determination of irrigation needs [19], the construction of mosaics of fields for weeds detection [20] and the support of WSNs in crop monitoring [21]. This last article can be considered as a previous step of this paper.

Although the use of UAVs is growing in outdoor farming, it is still limited in indoor farming. There are several tasks in greenhouse agriculture that could be performed using mini-UAVs: the measurement of climate variables, the monitoring of plants and the surveillance of the perimeter. Thus, despite of their current limitations (*i.e.*, autonomy, payload capacity and safety), their wide range of applications, low cost, versatility and precision augur a promising future for UAVs in indoor farming [22].

This paper presents a quadrotor-based sensory system for measuring environmental variables in a greenhouse. Aspects related to the navigation of the quadrotor in a restricted and irregular place are reflected in the bibliography [23]. Two challenges of this work, namely the quadrotor's limited payload and the possible influence of rotors on the sensors' measurements are successfully overcome.

## 2. System Overview

**Figure 1.**
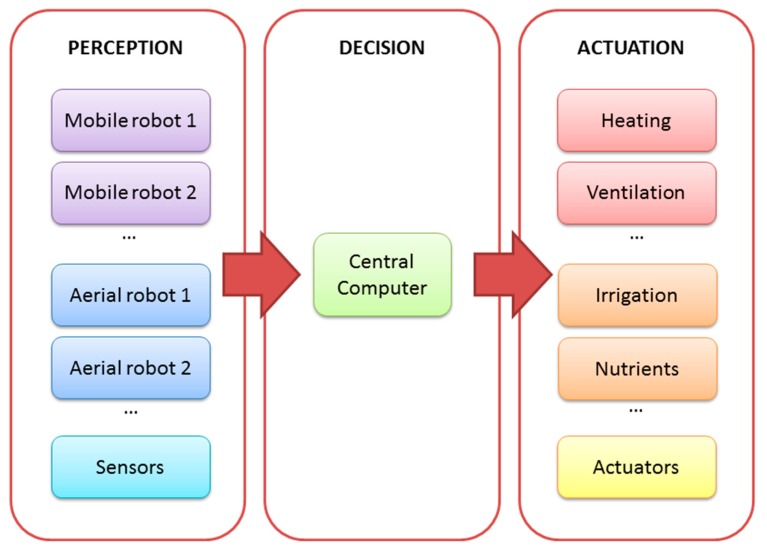
Architecture of the complete system.

The proposal of a mini-UAV-based sensory system is expected to be integrated in a greenhouse farming management system (Figure 1). The first one performs the acquisition of the environmental variables that can be measured in the air, while the second one encompasses not only the sensing (*i.e.*, acquisition of all environmental variables) but also the actuation (*i.e.*, climate control, crop monitoring and failure detection).

The complete system has a centralized architecture. A central computer receives data from sensing devices (e.g., ground or aerial robots and static sensors), compiles the data, makes decisions and sends commands to the actuation devices. The centralized architecture has advantages (e.g., all information is collected, managed and saved on a single computer) and weaknesses (e.g., the system cannot recover from a failure in the central computer).

In addition, the system has flexibility and modularity; it may be composed of different aerial and ground robots with different purposes (e.g., acquisition of air variables, determination of ground properties and supply of water, nutrients, fertilizers or protection products). The flexible and modular character of this system allows adding or removing robots to adapt to the needs of different greenhouses. All modules (*i.e.*, sensing, processing and effecting modules) and components (*i.e.*, aerial and ground robots, central computer, heating and ventilation systems, and other actuators) communicate via a wireless local area network.

However, one must remember that the aim of this work is the description of the quadrotor-based sensory system; this complete system is only the framework of this study. In the next subsections, a platform analysis together with the selection and integration of sensors are described.

### 2.1. Platform Analysis

The primary alternatives to the proposed mini-UAV sensory system are Wireless Sensor Networks (WSNs) and Unmanned Ground Vehicles (UGVs). Both of them are well-known solutions that have been applied in the context of greenhouse agriculture. Despite this fact, the WSNs and UGVs are hindered by limitations that UAVs can overcome.

The primary advantage of WSNs is simultaneous measurements at various points, which a UGV or UAV cannot perform and may be desirable for this application. WSNs are a robust solution due to their simplicity, which reduces the probability of failure, and their modularity, which allows working with damaged motes. Conversely, in contrast to UGVs and UAVs, WSNs are not able to move within the workspace to take measures at points of interest. In addition, the costs of WSNs strongly depend on the number of motes, which may reach hundreds in a medium size greenhouse. This multiplication of motes (e.g., sensors, controllers, batteries and communication modules) makes their costs higher than the costs of UGVs or UAVs.

UGVs tend to have lower costs than WSNs and are competitive against UAVs. The simplicity of their mechanic elements and control systems makes their costs lower than the costs of UAVs. In addition, UGVs can move to the points of interest; however, these movements are restricted to the ground, preventing them from reaching certain points of interest due to obstacles such as plants and covers. Conversely, UAVs can obtain measurements at nearly any point in a three dimensional space including at different altitudes. This fact is interesting not only for reducing the number of sensors and therefore the total cost of such a system but also for obtaining local data for production monitoring, problem detection (e.g., a break in a plastic cover) and local climate control. In summary, the characteristics of UAVs make them a competitive option for measuring the environmental variables of greenhouses and justify this research.

### 2.2. Selection of Sensors

Sensors have been selected based on the needs of climate control and crop monitoring activities. Multiple models of climate [24,25], temperature [26] and humidity [27] in greenhouses can be found in the literature. Additionally, a model of the growth and maturation of plants is available in [28]. The study of these models has determined the variables that should be measured; these include air temperature, air humidity, carbon dioxide concentration, ethylene concentration, ground temperature, ground humidity, nutrient concentration and solar radiation.

Among these variables, the ground temperature, ground humidity and nutrient concentration can be measured by a UGV with less risk and cost than by a UAV. Nevertheless, current ethylene sensors are too heavy to be placed on-board a quadrotor; thus, the incorporation of an ethylene sensor should be investigated in future works. This study will focus on demonstrating the capability of measuring gases using a mini-UAV this can be accomplished by testing and validating the use of a mini-UAV with a carbon dioxide concentration sensor.

Table 1 lists sensors for air temperature and humidity, carbon dioxide concentration and solar radiation measurement that are commercially available and their features. The final selection has been performed according to the criteria of weight, size, range, resolution and cost. Specifically, the RHT03 temperature and humidity sensor, and the MG811 carbon dioxide concentration sensor have been selected. The primary features of these sensors are shown in Table 2.

### 2.3. Integration of Sensors

The sensors have been integrated to satisfy two needs: the collection and storage of measurements including space and time references; and the communication between the mini-UAV sensory system and the greenhouse management system.

**Figure 2.**
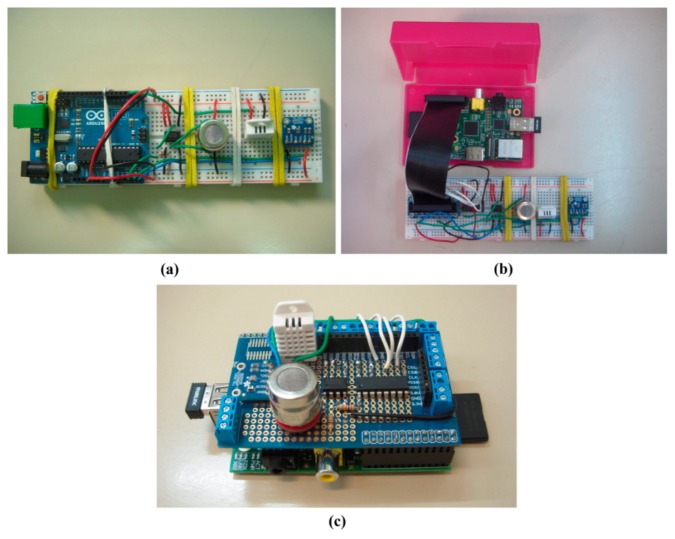
(**a**) Arduino UNO preliminary prototype; (**b**) Raspberry Pi preliminary prototype; (**c**) Raspberry Pi final prototype.

Several alternatives for the integration of sensors have been studied, and two prototypes have been developed: one with an Arduino UNO [29] (Figure 2a) and another with a Raspberry Pi [30] (Figure 2b). Both prototypes have been compared with multiple criteria including size, weight, performance and connectivity.

The Raspberry Pi has ultimately been chosen instead of the Arduino UNO due to its performance, connectivity and programming (Figure 2c). The Raspberry Pi has better performance than the Arduino UNO, both in hardware (e.g., processor speed and memory) and software (*i.e.*, operating system), which allows it to preprocess data while measuring. Additionally, the Raspberry Pi typically has better performance when connected to Wi-Fi networks and exchanging data with other devices. Finally, the Raspberry Pi provides additional programming capabilities including full integration with Robot Operating System (ROS) [31].

## 3. Location of Sensors

The location of sensors on the quadrotor is not a trivial issue and requires the study of some conditions. Both the air-flows produced by the rotors and the light and shadow conditions can affect the sensor measurements. Additionally, the weights of the sensors influence the weight and inertia of the quadrotor, which can in turn affect navigation.

Specifically, the temperature and humidity sensor can be affected by solar radiation and the airflows of the rotors, the luminosity sensor is obviously conditioned by solar radiation and the carbon dioxide sensor can be affected by the air-flows of the rotors.

Two previous studies have addressed quadrotor aerodynamics with similar results [32,33]. Their conclusions stated that when considering an isolated rotor, the airspeed is maximum at its perimeter and minimum in the center and the exterior of the quadrotor; moreover, considering all rotors, the airspeed is maximum near the rotors and minimum in the center and outside the quadrotor.

Based on the quadrotor aerodynamics and considering the effects of solar radiation, there are two possible sensor locations to consider: the center part of the top side of the quadrotor and outside the quadrotor at some distance. Considering both options, the first does not require a complex assembly that could modify the center of gravity of the quadrotor (e.g., an extension for the sensors) and therefore is selected for the location of the sensors. Unfortunately, the conclusions of both publications were focused on quadrotor design and modeling instead of sensor allocation. Therefore, a complementary exhaustive study of quadrotor aerodynamics oriented to sensor allocation was necessary.

Simulations of computational fluid dynamics (CFD) and real experiments for determining quadrotor airflows were performed to determine the relevant aerodynamics and validate the location of sensors. These simulations and experiments are described in Sections 3.1 and 3.2.

### 3.1. CFD Simulation of a Quadrotor

A set of CFD simulations has been performed to determine the aerodynamics of a quadrotor and the evolution of the airflows of the rotors. These simulations have been performed using Autodesk Simulation CFD 2014.

The simulation model of a quadrotor has been designed with maximum detail near the propellers to increase the precision of the results and lower complexity in the other parts of quadrotor to reduce the computational cost of simulations. The quadrotor designed has a wingspan of 400 mm (*i.e.*, the distance between opposite rotors) and its center is a square with sides 125 mm long.

These simulations have been performed in a transient regimen (*i.e.*, initially, the rotors were stopped but later accelerated up to 3000 rpm) instead of at steady state for two reasons: the range of speeds is wider, and these conditions are more unfavorable (*i.e.*, worst case scenario). In addition, the hypotheses of incompressibility and turbulence of airflows have been assumed because the rotation parts are small, and the airspeed is relatively low.

**Figure 3.**
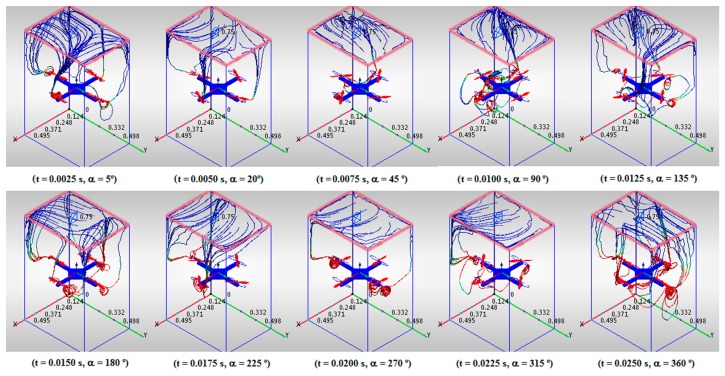
Airflows over the quadrotor.

**Figure 4.**
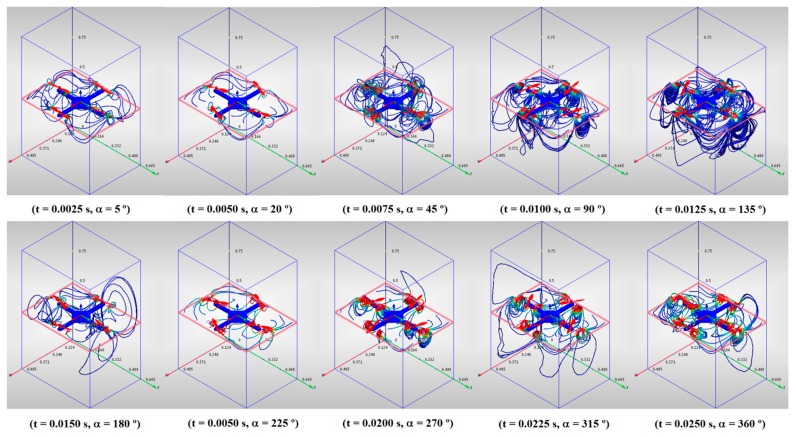
Airflows under the quadrotor.

**Figure 5.**
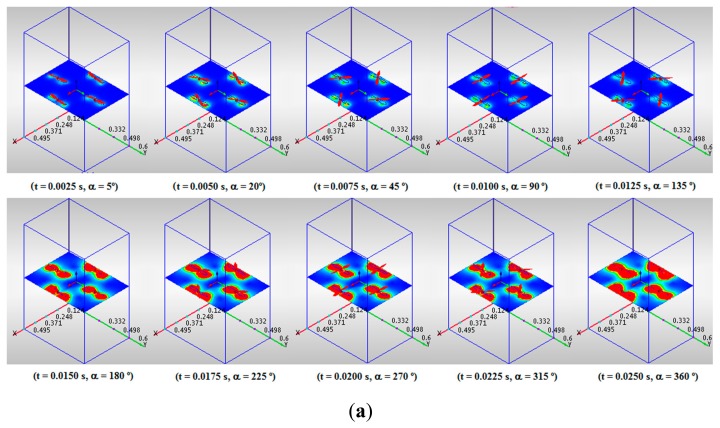

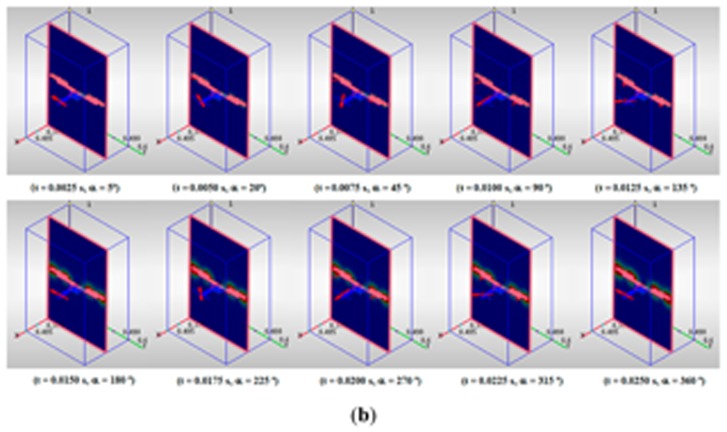
(**a**) Airspeed profile in the horizontal plane; (**b**) Airspeed profile in the vertical plane.

The CFD simulation results are shown in Figures 3, 4 and 5. The traces of fluid particles and the velocity profiles in planes have been chosen for a better visualization of the results to show them in a clear and precise manner from both a qualitative and quantitative perspective. Each frame is associated with its simulation time (t) and its propeller angle (α). The evolution of the airflows across the quadrotor can be seen in Figure 3. The traces arise from a plane located over the quadrotor and are attracted to the rotors. Their speed grows gradually as they approach the rotors and rapidly when they pass by them. Their trajectories follow the periphery of the air volume, avoiding the center of the quadrotor.

The evolution of the airflows under the quadrotor can be seen in Figure 4. The traces arise from a plane located immediately under the quadrotor and form a series of vortices around the rotors. Their speed grows rapidly when they pass near the rotors and falls gradually when they pass by them. As in the previous case, the center of the quadrotor is relatively free of airflows.

Figure 5 shows the airspeed profiles in the horizontal and vertical planes. As shown, the maximum speed is obtained within the rotors whereas the minimum speed is located near the center of the quadrotor. These results agree with the results of the previous works [32,33] and the hypotheses assumed in this work.

### 3.2. Measurement of Quadrotor Airflows

In order to determine the airspeed at different points around the quadrotor, an experiment has been performed to validate the results of the CFD simulations with a real quadrotor in real conditions.

**Figure 6.**
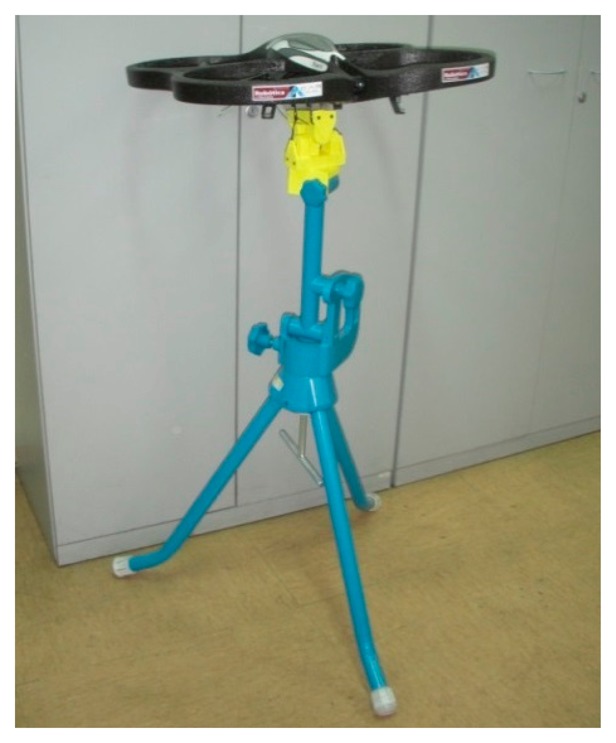
Layout of the experiment.

This experiment has been performed using the Parrot AR.Drone 2.0 quadrotor that is shown in Figure 6. The quadrotor was attached in a support with a Cardan joint, which allows its attitude to change while maintaining its location and altitude. This mechanism facilitated the experiment and reduced the risk of an accident. A grid of 24 positions located both under and over the quadrotor was defined to measure the air speed with a digital anemometer. Ten readings were registered at each grid point to calculate an average value at each grid point.

**Figure 7.**
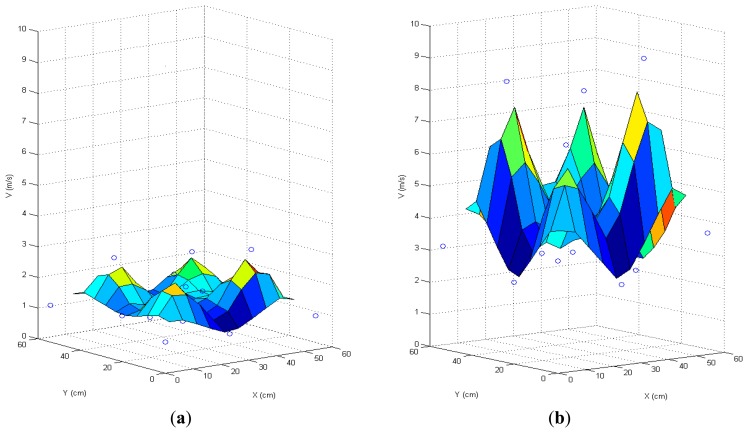
(**a**) Air speed over the quadrotor; (**b**) Air speed under the quadrotor.

Figure 7 depicts the results of this experiment. The airflows over the quadrotor are shown on the left side of the figure, and the airflows under the quadrotor are shown on the right. The *X* and *Y* axes show the location in centimeters, and the *Z* axis shows the airspeed in meters per second. The points are the measurements of the anemometer, and the surface is an interpolation of them.

The results of this experiment are coherent with the results of the CFD simulations. The airspeed presents four maxima within the four rotors and a depression in the center of the quadrotor. In addition, the results show that the velocity of the air repelled by the rotors (Figure 7b) is higher than the velocity of the air attracted by them (Figure 7a).

**Figure 8.**
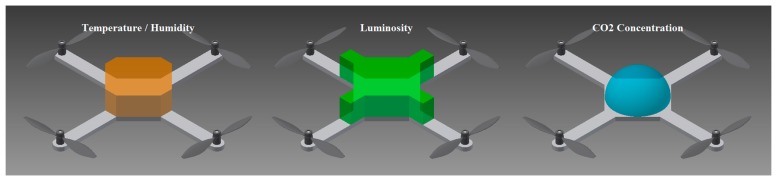
Proposal of sensor location.

Both the simulations and experiments confirm the hypothesis that the optimal location for the sensors is on the center of the top side of the quadrotor. A proposal of the most adequate areas for allocating the different sensors is shown in Figure 8.

### 3.3. Measuring on-Board a Quadrotor

The previous simulation and experiments have determined the optimal placement for the sensors in the quadrotor, but they have not provided any conclusions about the feasibility of performing measurements with the sensors in this location. Therefore, an additional experiment has been conducted to validate that the rotors' airflows have no significant influence in the sensors' measurements. This experiment consisted of taking measurements at a series of points under two different conditions: with the propellers stopped and with the propellers active.

Three sources of temperature, humidity and carbon dioxide were used to create gradients of these variables in the workspace. As in the previous experiment, a Parrot AR.Drone 2.0 quadrotor was used to transport the sensor system.

**Figure 9.**
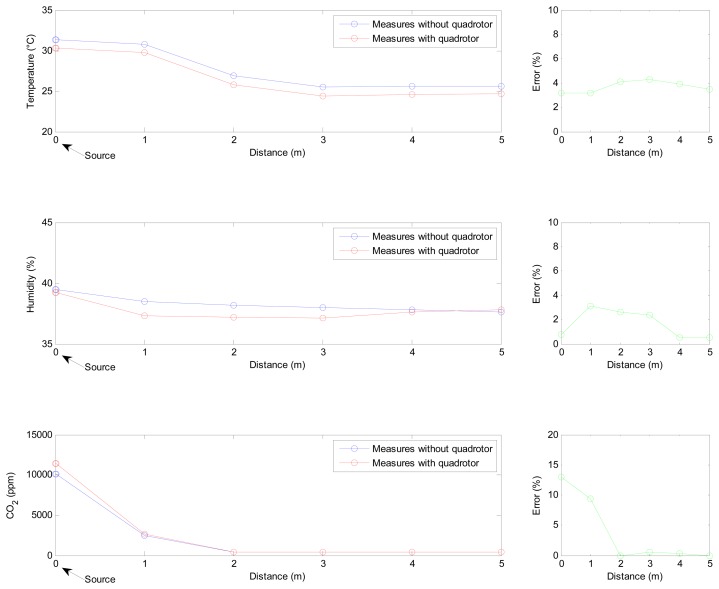
Temperature, humidity and CO_2_ measured with propellers stopped and working.

Measurements were taken from a distance of 5 m to the sources at intervals of 1 m. In the first test, the quadrotor was moved by hand, and in the other, it flew autonomously. In both experiments, the quadrotor was at a height of 0.5 m, and the sources were located on the ground. The results of this experiment are shown in Figure 9. As can be seen, there are differences between the measurements obtained with the rotors stopped and when they are moving. However, the average relative errors in temperature (3.71%), humidity (1.65%) and carbon dioxide concentration (3.84%) can be considered to be negligible. These errors can be associated with multiple factors apart from the influence of the propellers, including possible changes in the environment over time, particularly with regard to temperature and humidity, and the response time of the sensors, particularly with regard to carbon dioxide.

## 4. Experiments, Results and Discussion

In order to validate the developments made in the laboratory, a series of field experiments was carried out in a greenhouse located in Almeria (Andalucia, Spain), an area with massive use of greenhouse farming.

In these experiments, maps of temperature, humidity, luminosity and carbon dioxide concentration in a greenhouse have been built using the mini-UAV based sensory system. The quadrotor followed a pre-planned path to avoid collisions with obstacles and obtained its location using visual odometry (*i.e.*, following a line and stopping at squares printed on the ground) and measurements from its Inertial Measurement Unit (IMU). In order to avoid possible errors due to the response times of the sensors, the quadrotor stopped at the waypoints until their measurements were stable.

The greenhouse was rectangular (106 m × 47 m) and had a height of 3 m; there were two doors on the front side of the building and two windows along its roof. The experiments were performed on 2 June 2014, starting at 9:00 a.m. and finishing at 10:00 a.m. During the experiments, the greenhouse was fallow, a tractor was working inside, and the doors and windows were open for ventilation.

**Figure 10.**
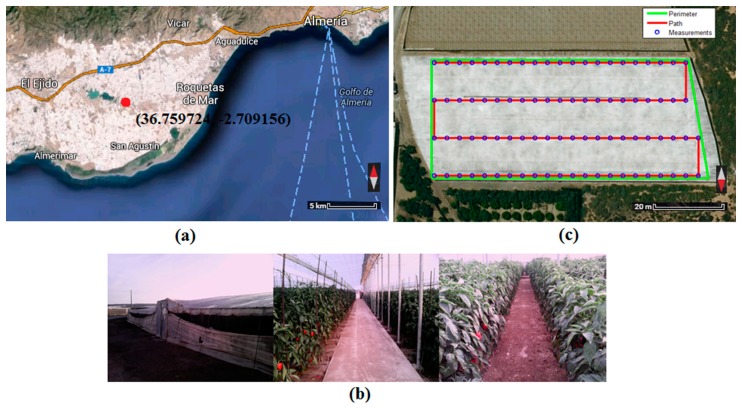
(**a**) The “sea of plastics” in Almería (Andalucía, Spain). Map data ©2014 Google, based on BCN IGN Spain; (**b**) Inside and outside of the greenhouse; (**c**) Top view of the greenhouse. Map data ©2014 Google, based on BCN IGN Spain.

**Figure 11.**
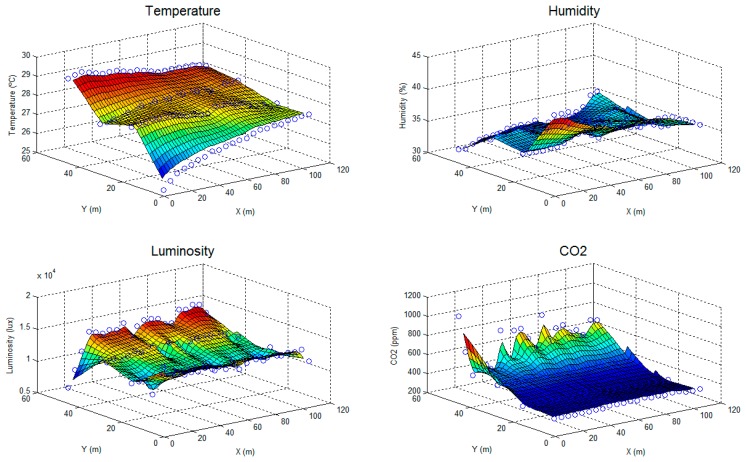
Maps of temperature, humidity, luminosity and CO_2_ concentration of the greenhouse.

Different perspectives of the greenhouse (e.g., outside, inside, front and top) are shown in Figure 10. The covered surface, the measurement points and the path followed in the experiments are detailed in the top view (Figure 10c). The maps of temperature, humidity, luminosity and carbon dioxide concentration obtained in the greenhouse are shown in Figure 11. The points show the measurements of the sensors, and the surfaces show interpolations between these points.

As shown, the temperature grew from the first measurement (25.3 °C), located at (1,1), to the last measurement (29.6 °C), located at (46,1). This fact is explained by considering the time differential of these measurements, which began at 9:00 and ended at 9:22; these measurements corresponded to the transition between nighttime and daytime temperatures and the overall warming of the greenhouse.

In contrast, the humidity declined from 43% to 33% from the beginning to the end of the experiment at the same locations as the temperature measurements. This behavior is also justified by considering the differences between nighttime and daytime humidities. Additionally, the values of humidity (e.g., 30%–50%) were lower than is typical in greenhouses (e.g., 70%–90%); this was likely because the greenhouse was not in production during these experiments, and a supply of humidity from the evapotranspiration of plants was absent.

The luminosity map is shown to be more regular, but it presents some shadowed locations. It is noticeable that the greenhouse cover filters luminosity: the sensor measurements were approximately 40,000 lux outside and 14,000 lux inside.

Finally, the carbon dioxide concentration shows spatial variation; specifically, this variable increased more in the *Y*-axis, which was likely due to the tractor mentioned before, which was working in that area, and the ventilation, which was worse on that side of the greenhouse.

**Figure 12.**
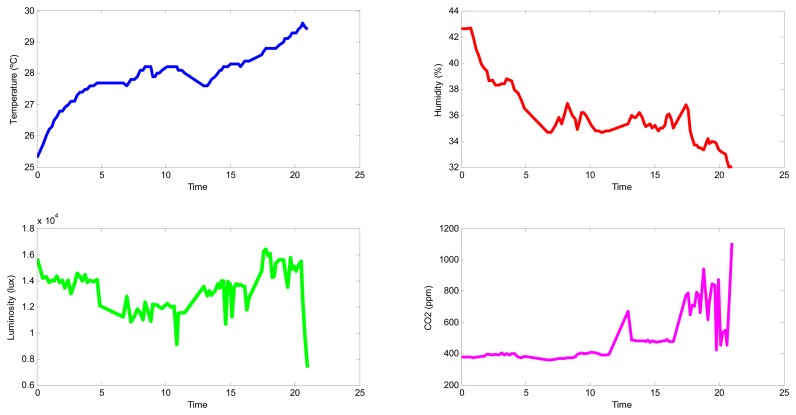
Variable dependence with time.

The expected correlation among some variables and time is shown in Figure 12. This fact highlights one of the limits of the mini-UAV based sensory system—its inability to take simultaneous measurements at different points. However, when considering a steady state, the coverage time required is small enough to still monitor the complete greenhouse and obtain valuable information. Depending on the size of the greenhouse, a fleet of mini-UAVs instead of a single mini-UAV could be used to obtain more homogeneous measurements and build maps more efficiently.

The results of these experiments demonstrate that the mini-UAV sensory system is able to measure the temperature, humidity, luminosity and carbon dioxide concentration in a greenhouse, allowing maps of the distribution of these variables to be built, and to capture the spatial and temporal variation of these variables.

## 5. Conclusions

This paper proposes a quadrotor-based sensory system for measuring environmental variables of a greenhouse. In contrast to Wireless Sensor Networks (WSNs), Unmanned Ground Vehicles (UGVs) and other solutions, Unmanned Aerial Vehicles (UAVs) are able to obtain measurements at nearly any point in the three dimensional space of the greenhouse, which facilitates activities such as local climate control and crop monitoring. The primary contributions of this paper are the determination of the optimal location of sensors on the quadrotor and the validation of a quadrotor as a platform for measuring environmental variables.

First, an exhaustive study of quadrotor aerodynamics was performed in order to determine the optimal allocation for sensors in the quadrotor. This study was supported by Computational Fluid Dynamics (CFD) simulations and experiments and has concluded that the optimal location for the sensors is the central part of the top side of the quadrotor. The results of this study can be applied to different contexts, including the design of a high-efficiency quadrotor and the location of other sensors and actuators.

Second, a set of field experiments was performed in a greenhouse to validate the mini-UAV sensory system. These experiments have shown that the system can collect the environmental variables of the greenhouse, including the gas concentrations together with their spatial and temporal variability and possible disturbances. Differences in the sensor measurements that can be attributed to the rotors' influence were bounded; relative errors were lower than 4%. The system allows for climate control, crop monitoring and failure detection in a greenhouse and can be implemented in other industries and infrastructures. Finally, the system can incorporate other sensors for measuring other gases such as CO, CH_4_, SO_2_ or NO_2_, if required.

## Figures and Tables

**Table 1.** Analysis of environmental variables.

**Variable**	**Is It Better to Measure It in the Air or on the Ground?**	**Is There a Sensor That Can be Attached to a Mini-UAV?**	**Result**
Air temperature	√	√	√
Air humidity	√	√	√
CO_2_ concentration	√	√	√
Ethylene concentration	√	×	×
Ground temperature	×	√	×
Ground humidity	×	√	×
Nutrient concentration	×	×	×
Solar radiation	×	√	√

**Table 2.** Sensor features. Source: Sensor datasheets.

**Features**	**Sensors**		

	**Temperature/Humidity: RHT03**	**Luminosity: TSL2561**	**CO_2_** **Concentration: MG811**
Power supply	3.3–6.0 V	2.7–3.3 V	6.0 V

Measurement range	T: [−40; 80] °C	[0; 40,000] lux	[350; 10,000] ppm
	H: [0; 100]%		

Sensitivity	T: 0.1 °C	1 lux	Variable
	H: 0.1%		

Accuracy	T: 0.5 °C	Not available	Not available
	H: 2%		

Preparation time	0–5 s	0–1 s	30–60 s

Response time	0–5 s	0–1 s	15–30 s

Communications	Digital	I2C	Analog
